# Ultrasonography Findings of Testicular Tuberculosis in Nepalese Patients: A Case Series

**DOI:** 10.31729/jnma.8835

**Published:** 2024-12-31

**Authors:** Madan Thapa, Sushmit Kafle, Pratik Lamichhane, Suresh Thapa

**Affiliations:** 1 Department of Radiology, Pokhara Academy of Health Sciences, Pokhara, Kaski, Nepal; 2 Department of Radiology, GP Koirala National Center for Respiratory Diseases, Belchautara, Tanahun, Nepal; 3 Department of Medicine, GP Koirala National Center for Respiratory Diseases, Belchautara, Tanahun, Nepal

**Keywords:** *diagnosis*, *testis*, *tuberculosis*, *ultrasonography*

## Abstract

Extrapulmonary tuberculosis constitutes about 20% of all cases of tuberculosis. It involves organs other than the lungs, such as lymph nodes, the genitourinary tract, abdomen, skin, joints and bones, and meninges. Tuberculosis of the testis is a rare disease. The diagnosis of testicular tuberculosis could be confused with testicular cancer, sarcoidosis or metastases. Herein, we describe the ultrasonography of five patients with testicular tuberculosis. This case series highlights the importance of ultrasonography in the diagnosis of rare form of extra pulmonary tuberculosis.

## INTRODUCTION

Tuberculosis (TB) is an infectious disease caused by the Mycobacterium bacteria with the EPARa potential to affect multiple organ systems.^1^ Extrapulmonary tuberculosis (EPTB) is a less common form of TB and affects lymph nodes, pleura, bones and joints, meninges, and genitourinary (GU) tract.^2^ Among GU-TB, testicular TB is extremely rare, representing only about 3% of GU-TB.^3,4^ The common presentation of a testicular TB is a painless or slightly painful scrotal mass. The differential diagnosis of such a scrotal mass includes testicular tumor, acute infection, infarction, and granulomatous infection When clinical findings mimic those of a tumor, the diagnosis of TB is likely to be missed because tumors are a more common cause of scrotal mass.^5^ Here, we present ultrasonography(USG) findings of fases of with TB infection of the testis and epididymis. An informed consent was obtained from all the participants involved in this case series.

## CASE 1

**Figure 1:**
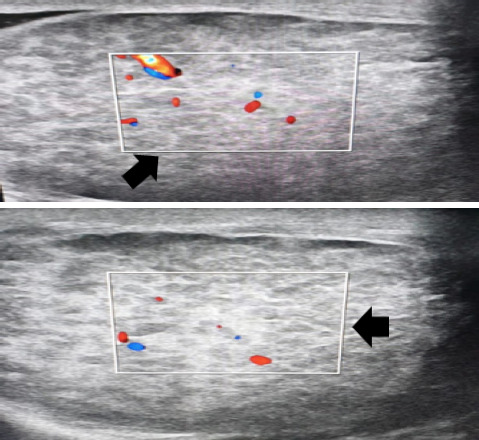
Multiple uniform size well-defined hypoechoic lesions are noted in right testis.

A 20 years male presented with persistent pubic pain of unknown origin for a two and a half months. Incidentally, right testicular mass was detected on physical examination. The mass was elliptical, 2.5cmx2cm in size, hard, tender, non-pulsatile, and not attached with the scrotal skin. The overlying skin was also unremarkable. The ultrasonography (USG) revealed multiple well-defined hypoechoic lesions on right testicle (Figure 1). The diagnosis was further confirmed with the with Ziehl-Neelsen staining of aspirate obtained from affected testes with FNAC technique.

## CASE 2

A 35-years male presented with a history of left testicular swelling for four months. The patient also complained of dry cough and fever during night time for three months. He also gave a history of similar swelling the right testis 15 days back. Upon investigation, ultrasonography of scrotum showed numerous uniform-sized well-defined hypoechoic lesions in bilateral testis (Figure 1). The fine needle aspiration of affected testes was obtained which was stained with Ziehl-Neelsen staining which revealed acid-fast bacilli (AFB). Likewise, the GeneXpert test of the sputum sample of the patient also came out positive. Hence, the patient was diagnosed with pulmonary tuberculosis and concomitant testicular tuberculosis. The patient is currently on anti-tubercular therapy (ATT) after confirmation of the diagnosis.

## CASE 3

**Figure 2:**
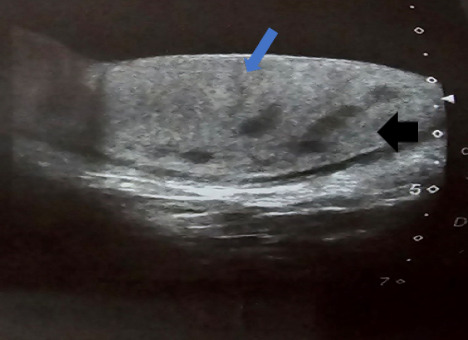
Multiple well defined hypoechoic lesions noted in testis with mild fluid in the scrotal sac (hypoechoic lesions of testicular tuberculosis (pointed by black solid arrow) and fluid in scrotal sac (pointed by blue solid arrow).

A 42-years male presented with the complaints of painless swelling in the right testis for three months. The swelling was round, pea-sized, smooth texture, non-mobile, non-pulsatile, non-tender, and hard in consistency. There were no changes in the overlying skin of the scrotum. He was diagnosed with pulmonary TB about two month ago and was taking ATT for 28 days at the time of USG. The USG revealed multiple well-defined hypoechoic lesions noted in testis with mild fluid in the scrotal sac (Figure 2).

## CASE 4

**Figure 3:**
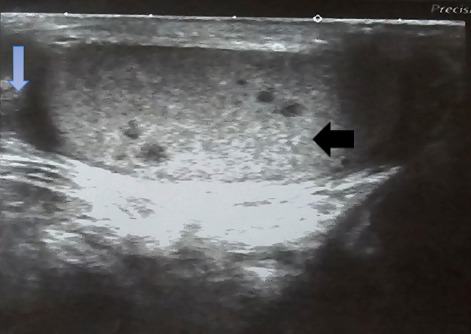
Multiple hypoechoic lesions noted in testis (pointed by black arrow) and thickened and edematous spermatic cord (pointed by blue arrow).

A 33-years male patient presented with the complaints of painless right testicular swelling for two weeks. The swelling was round, 2cmx1cm in dimension, non-tender, hard, non-pulsatile, and non-mobile. The overlying skin of the scrotum was unremarkable. The patient was bacteriologically confirmed case of pulmonary TB. At the time of USG, he was on ATT for four weeks. His USG shows multiple hypoechoic lesions noted in testis and thickened and edematous spermatic cord (Figure 3). The diagnosis of testicular TB was confirmed later on with the FNAC and Ziehl-Neelsen staining.

## CASE 5

A 19-years male presented with complaint of longstanding unilateral testicular pain and diffusely enlarged swelling for a year. The pain was dull in nature, intermittent, localized to left testes, nonradiating, and mild in intensity. The patient had no urinary tract-related symptoms. The patient had a history of bacteriologically confirmed TB with acid-fast staining and had been on ATT for a month. The diagnosis of testicular TB was later confirmed using fine needle aspiration cytology technique. The aspirate showed AFB with Ziehl-Neelsen staining. The USG showed multiple well-defined hypoechoic lesion noted in testis (USG findings similar as in Figure 1).

## DISCUSSION

Tuberculosis can affect virtually any organ system in the human body, excluding hairs and nails. Even though pulmonary TB is the most common form of TB, EPTB poses a significant burden on the public health. Testicular TB is an uncommon condition, accounting for only around 3% of all instances of GU-TB.^6^ Given the rarity of solitary testicular involvement in EPTB, it is important to consider conditions such as testicular tumor, infarction, and other granulomatous infections when making a differential diagnosis.^7^ There is a possibility of testicular TB being misdiagnosed as a testicular tumor since both conditions can present with a testicular mass, pain, and swelling. Subsequently, the misdiagnosis can lead to an increase in the number of unnecessary orchiectomies.^8^

The epididymis is the most common primary location for TB infections in the scrotum. Tubercular orchitis typically occurs when the infection spreads from the epididymis to the testicles. The occurrence of orchitis without involvement of the epididymis is uncommon, but it can happen through the transfer of infection through the bloodstream.^4^ The initial occurrence of tubercular epididymitis is a result of the early involvement of mycobacteria spreading by urine reflux in a retrograde manner. Furthermore, the tail of the epididymis receives a more substantial blood supply, which could perhaps contribute to its early involvement. The simultaneous occurrence of epididymis involvement and testicular lesion supports the diagnosis of infection.^9^

Testicular biopsy is the gold standard of diagnosis of testicular TB. Fine needle aspiration biopsy (FNAB) and cytology can show epithelioid granulomas, and bacilli can be detected by acid-fast staining. However, AFB staining is not always confirmatory, AFB positive results were detected in only about 60% of tuberculosis cases.^10^ The USG can be a valuable screening and diagnostic test for patients who are suspected to have testicular TB. On the basis of USG findings, testicular TB can be categorized into four different types: (a) diffusely enlarged with heterogeneously hypoechoic lesions ; (b) diffusely enlarged testes with homogenously hypoechoic lesions; (c) nodular enlargement of testes with heterogeneously hypoechoic lesions; and (d) miliary involvement of testes. The commonly observed USG findings in testicular TB includes diffuse testicular size, heterogeneous or homogeneous hypoechoic nodular lesions can be observed on USG.^11,12^ Four out of five of our cases had a diffusely enlarged testicular swelling with multiple homogenous or heterogeneous hypoechoic lesions. However, one of the cases had a nodular enlargement of unilateral testes with multiple heterogeneously hypoechoic lesions. Likewise, one of the cases showed an additional involvement of spermatic cord with edema and thickening. Furthermore, color doppler USG can be employed to distinguish testicular TB from other vascular pathologies such as torsion, infarcts, and tumors.^13^ Hence, USG is considered the investigation of choice for diagnosis of testicular TB.^5^

Some conditions like sarcoidosis, testicular lymphoma, primary testicular tumors, infarcts, and infections mimic testicular TB and could be difficult to diagnose with USG alone. Due to vague sonographic patterns, imaging features are often non-specific and difficult to distinguish TB from other inflammatory causes, tumor, or infarction.^5^ Sarcoid granulomas can be observed on USG as either solitary or multiple hypoechoic nodules within the testes, resembling TB. In sarcoidosis, testicular involvement without epididymitis is infrequent. However, the presence of concomitant systemic signs of sarcoidosis can serve as a valuable indicator for diagnosing sarcoidosis.^14^ Likewise, lymphoma usually presents as diffuse enlargement of testes with large hypoechoic infiltrative area replacing the testes but characteristically maintaining normal testicular shape. Testicular tumors and other non-tumorous diseases, such as localized infarct, hematoma, and infection, might have similar imaging characteristics. These include areas that are hypoechoic and have varying blood flow. A testicular mass with internal vascularity should be presumed to be a testicular tumor until shown otherwise.^14^ Color doppler imaging can aid in distinguishing between infarction, malignancy, and inflammation. Tubercular epididymitis and orchitis can be identified on color doppler by the presence of peripheral vascularity caused by granulomas and the absence of central flow due to caseation necrosis. This is in contrast to tumors, which typically exhibit central vascularity.^14^

The majority of the cases had symptoms such as fever, night sweats, unexplained weight loss in their medical history, a young age, and the involvement of only one testicle, which strongly indicated testicular TB. The caeseified necrotizing granuloma mentioned in the FNAB report was considered a conclusive diagnosis in our instances, and therapy was subsequently determined based on this diagnosis. Furthermore, the patient's symptoms showed recovery after six months of ATT, and the nodules on the testicular USG disappeared, which further supported our diagnosis of testicular TB. While surgical intervention is seldom necessary due to complications, testicular tuberculosis can be adequately treated with a quadruple regimen of isoniazid, rifampicin, pyrazinamide, and ethambutol, administered for a duration of six months.^15^

Testicular TB should definitely be considered in the presence of unilateral heterogeneous mass, especially in young men with a history of living or traveling in a TB endemic region. In resource-constrained like ours, USG can be a valuable imaging technique for screening and diagnosis of testicular TB.
